# Safety of Pediatric HIV Elimination: The Growing Population of HIV- and Antiretroviral-Exposed but Uninfected Infants

**DOI:** 10.1371/journal.pmed.1001636

**Published:** 2014-04-29

**Authors:** Lynne M. Mofenson, D. Heather Watts

**Affiliations:** 1 Eunice Kennedy Shriver National Institute of Child Health and Human Development, National Institutes of Health, Bethesda, Maryland, United States of America; 2 Office of the Global AIDS Coordinator, US Department of State, Washington (D.C.), United States of America

## Abstract

Lynne Mofenson and Heather Watts discuss the context and implications of the study by J. Sibuide and colleagues, which provides a detailed analysis of birth defects in infants with in utero antiretroviral drug exposure in the French Perinatal Cohort.

*Please see later in the article for the Editors' Summary*

**Linked Research Article**

This Perspective discusses the following new study published in *PLOS Medicine*:

Sibiude J, Mandelbrot L, Blanche S, Le Chenadec J, Boullag-Bonnet N, et al. (2014) Association between Prenatal Exposure to Antiretroviral Therapy and Birth Defects: An Analysis of the French Perinatal Cohort Study (ANRS CO1/CO11). PLoS Med 11(4): e1001635. doi:10.1371/journal.pmed.1001635

Jeanne Sibiude and colleagues use the French Perinatal Cohort to estimate the prevalence of birth defects in children born to HIV-infected women receiving antiretroviral therapy during pregnancy.

One of the greatest public health successes has been the development and implementation of antiretroviral interventions to prevent mother-to-child HIV transmission (MTCT). The landmark 1994 Pediatric AIDS Clinical Trials Group (PACTG) 076 results, demonstrating zidovudine given to HIV-infected pregnant women and their infants reduced MTCT by nearly 70%, led to rapid implementation in the Unites States, with subsequent decline in MTCT from 25% to 4%–5% within two years [1]. Pregnant HIV-infected women in the United States and other high-resource countries now receive combination antiretroviral therapy (cART) including three or more drugs, which has led to further reductions in MTCT; in the United States, MTCT has been nearly eliminated, with rates currently <2% [2].

Additionally, there has been striking progress in reducing MTCT in resource-constrained countries, with 1 million children prevented from acquiring HIV between 2003 and 2013 because of maternal and infant antiretroviral prophylaxis [3]. The 2013 WHO consolidated guidelines recommend that all HIV-infected pregnant women initiate cART, and if breastfeeding, continue cART throughout breastfeeding [4]. After the MTCT risk period has ended, women may either continue life-long treatment regardless of clinical status or stop if they do not meet treatment eligibility criteria for non-pregnant individuals.

However, accompanying this success is a rapidly expanding population of HIV-exposed but uninfected children with substantial exposure to antiretroviral drugs, both in utero and, in resource-constrained countries, while breastfeeding. There is an urgent need to better understand the consequences of antiretroviral drug exposure on HIV-uninfected children and to improve monitoring and management of any potential adverse effects in this burgeoning population. Sibiude and colleagues in this week's issue of *PLOS Medicine* provide a detailed analysis related to birth defects in infants with in utero antiretroviral drug exposure in the French Perinatal Cohort [5].

Given the emphasis on early treatment of HIV in adults and the move toward initiation of life-long therapy in all pregnant women in many resource-constrained countries, it can be anticipated that there will be a dramatic increase over time in women who conceive while receiving antiretroviral drugs, with fetal exposure from conception onward. Data from epidemiologic studies suggest MTCT may be lowest in infants born to mothers receiving cART prior to conception and continued during pregnancy [6]. However, there are only limited data on potential toxicities of fetal/infant antiretroviral drug exposure.

## Birth Defects and Drug Exposures

A critical factor in the risk of drug-related birth defects is fetal developmental stage at the time of exposure. During the first two weeks after conception, exposures are unlikely to cause malformations, as immediately after conception the embryo has not yet formed, and after its formation, an additional period of time intervenes before its cells become committed to specific developmental paths [7]. The time of greatest sensitivity to teratogenic exposures is the stage of organogenesis (18–60 days after conception or 4–13 weeks after the beginning of the last menstrual period), before many pregnancies are recognized, particularly in resource-constrained settings. Exposures later in gestation are less likely to produce gross structural abnormalities of the fetus. However, because this period is a time of active cell growth, differentiation, maturation, and migration, particularly in the central nervous system (CNS), teratogenic exposures may cause growth retardation or functional CNS disorders that may not be apparent until much later in life and would be missed by examination confined to the neonatal period.

Animal studies have been used to evaluate whether drug exposures may be associated with teratogenic potential prior to use in humans. However, it is difficult to extrapolate animal findings because of species differences in placentation, embryonic development, innate predisposition to fetal abnormalities, and drug pharmacokinetics/dynamics [7]. Thus, identification of human teratogenic exposures relies on epidemiologic studies, but such studies require careful interpretation. Ascertainment and recall biases can result in erroneous associations. Additionally, the maternal disease that created the need for administration of the drug rather than the drug itself could be responsible for an observed association. Teratogenic exposures most often produce distinct or characteristic patterns of congenital abnormalities as opposed to a single malformation in an otherwise normal child [7]. Importantly, a statistically significant association in an epidemiologic study does not necessarily indicate causality.

## Birth Defects with Antiretroviral Drug Exposure: French Perinatal Cohort

The French Perinatal Cohort study reports on birth defect surveillance in 13,124 live-births to HIV-infected pregnant women delivering in 90 clinical centers in France between 1994 and 2010; 5,388 (42%) of infants had first trimester exposure to one or more antiretroviral drugs [5]. There are a number of important differences between the French Perinatal Cohort and most other reports on birth defects, including the Antiretroviral Pregnancy Registry [8]. In France, fetal ultrasounds in each trimester are standard-of-care for all pregnant women, and the identification of birth defects in the children in their cohort extended through age two years. In contrast, the Antiretroviral Pregnancy Registry and many published studies report on defects detected at birth or within a few days of birth, without extended follow-up for later detection of birth defects, and active fetal ultrasound surveillance was not conducted.

The primary finding in the the French Perinatal Cohort study was a significant association of first-trimester zidovudine exposure with congenital heart defects, which persisted after adjustment for a number of potential confounders. Most were ventricular or atrial septal defects (58% and 18%, respectively) and persistent ductus arteriosus, and were not associated with other malformations. The clinical significance of these defects or whether these were primarily detected through active fetal ultrasound surveillance was not described. Spontaneous closure of ventricular septal defects (VSDs) is frequent; in a study of 249 fetuses with VSDs detected by fetal ultrasound, spontaneous closure of the VSD occurred in 5% of fetuses prenatally and 76% postnatally by age one year [9]. The association of heart defects with first trimester zidovudine exposure has been reported in two smaller studies but not in larger cohorts, including the Antiretroviral Pregnancy Registry [8],[10],[11].

The authors also report a significant association between first-trimester efavirenz exposure and neurologic defects in the MACDP classification system of birth defects, but not the EUROCAT classification, which excludes minor anomalies with no serious medical or functional consequences [12]. Efavirenz-based cART is the WHO recommended regimen for pregnant HIV-infected women; primate data have raised concerns regarding potential CNS teratogenicity although data in humans have been more reassuring [4],[13]. The MACDP association was based on only four CNS defects, two of which (subependymal cyst and partial agenesis of the corpus callosum) are asymptomatic findings likely detected on fetal ultrasound that may have no clinical significance. None were neural tube defects (a defect of concern from primate studies) and they do not have similar embryologic origins. Interestingly, the three neural tube defects (spina bifida) noted in live-births in the study were not in efavirenz-exposed infants. While drug exposure for the two neural tube defects observed in pregnancy terminations was not specified, since sensitivity analyses including these defects were performed, it is assumed these were also not in efavirenz-exposed infants. Thus, these data are actually reassuring regarding a lack of neural tube defects in infants with first-trimester efavirenz exposure [5].

Reassuringly, no association with birth defects were observed for other WHO-recommended first or second-line drugs, including tenofovir, lamivudine, and lopinavir/ritonavir [5].

## Clinical Implications of the French Perinatal Cohort Data

The use of antiretroviral drugs by HIV-infected pregnant women has resulted in a paradigm shift in the maternal and pediatric HIV epidemics, with markedly improved maternal survival and dramatic reductions in MTCT. However, despite the widespread use of antiretroviral drugs by pregnant women, systematic evaluation of birth defects has been limited. The Antiretroviral Pregnancy Registry only enrolls ∼15% of HIV-infected pregnant women giving birth annually in the United States and only about 200 pregnant women from other countries [8]; clinicians are urged to report exposures to the Registry.

The largest amount of data on birth defects and antiretroviral drug exposure comes from high-resource countries. However, the largest population of HIV-infected women of childbearing age resides in resource-constrained countries, where women are exposed to multiple factors that could increase risk of birth defects, such as under-nutrition, micronutrient deficiency, anemia, and co-infections. Additionally, in many resource-constrained countries, data on the background risk of birth defects in the general population are lacking. Determining the potential risk of birth defects due to antiretroviral drug exposure requires knowledge of the underlying risk of birth defects in the population being studied.

Given that the HIV burden in women is greatest in resource-constrained countries, where rapid expansion in antiretroviral drugs use in pregnancy (and increasingly at conception) is expected with implementation of WHO guidelines, there is an ethical imperative to systematically and critically evaluate the safety of these recommendations for the fetus/infant, both in terms of potential teratogenicity but also long-term outcomes. While the Sibiude study raises some important questions, given the enormous benefits of maternal antiretroviral drugs, the unclear clinical significance of the heart defects and the lack of a specific pattern of CNS defects with efavirenz, no change in prescribing practices is indicated, but continued surveillance is critical.

WHO has established a Pregnancy Registry protocol that is beginning to be implemented in several resource-constrained countries [14]. Additionally, the President's Emergency Plan for AIDS Relief (PEPFAR) is initiating active surveillance for birth defects in HIV-infected and -uninfected women at sentinel sites in Malawi and Uganda. These data will be critical in evaluating safety of cART regimens and determining the best regimens to ensure the greatest benefit to the health of both the mother and her child.

## Abbreviations

cART: combination antiretroviral therapy
CNS: central nervous system
MTCT: mother-to-child HIV transmission
